# The influence of radio frequency ablation on intra-articular fluid temperature in the ankle joint - a cadaver study

**DOI:** 10.1186/s12891-018-2347-5

**Published:** 2018-11-24

**Authors:** Philipp Ahrens, Dirk Mueller, Sebastian Siebenlist, Andreas Lenich, Ulrich Stoeckle, Gunther H. Sandmann

**Affiliations:** 1 Department of Orthopaedic Sports Medicine, Klinikum rechts der Isar, Technische Universitaet Muenchen, Germany, Ismanninger, Str. 22, D- 81675 Muenchen, Germany; 2 Sportklinik Stuttgart, Taubenheimstraße 8, D-70372 Stuttgart, Germany; 3 Schön Klinik Harthausen, Dr.-Wilhelm-Knarr- Weg 1-3, D-83043 Bad Aibling, Germany; 4 Helios Klinikum München West, Steinerweg 5, D- 81241 Muenchen, Germany; 5 BG Unfallklinik Tuebingen, Schnarrenbergstraße 95, 72076 Tuebingen, Germany; 6 Sportklinik Ravensburg, Bachstraße 57, 88214 Ravensburg, Germany

**Keywords:** Ankle joint, Radio frequency, Ablation, Thermal damage, Chondrocytes

## Abstract

**Background:**

Radio frequency ablation devices have found a widespread application in arthroscopic surgery. However, recent publications report about elevated temperatures, which may cause damage to the capsular tissue and especially to chondrocytes. The purpose of this study was the investigation of the maximum temperatures that occur in the ankle joint with the use of a commercially available radio frequency ablation device.

**Methods:**

Six formalin-fixed cadaver ankle specimens were used for this study. The radio frequency device was applied for 120 s to remove tissue. Intra-articular temperatures were logged every second for 120 s at a distance of 3, 5 and 10 mm from the tip of the radio frequency device. The irrigation fluid flow was controlled by setting the inflow pressure to 10 mmHg, 25 mmHg, 50 mmHg and 100 mmHg, respectively. The controller unit voltage setting was set to 1, 5 and 9.

**Results:**

Maximum temperatures exceeding 50 °C/122 °F were observed for all combinations of parameters, except for those with a pressure of 100 mmHg pressure. The main critical variable is the pressure setting, which is highly significant. The controller unit voltage setting showed no effect on the temperature measurements. The highest temperature was 102.7 °C/215.6 °F measured for an irrigation flow of 10 mmHg. The shortest time span to exceed 50 °C/122 °F was 3 s.

**Conclusion:**

In order to avoid temperatures exceeding 50 °C/122 °F in the use of radio frequency devices in arthroscopic surgeries of the ankle joint, it is recommended to use a high irrigation flow by setting the pressure difference across the ankle joint as high as feasible. Even short intervals of a low irrigation flow may lead to critical temperatures above 50 °C/122 °F.

**Level of Evidence:**

Level II, diagnostic study.

## Background

Arthroscopic surgery of the ankle joint is widespread and tissue ablation is one of the main indications in the “Soccer’s Ankle” or anterior impingement. In this condition, fibrous tissue causes pain above the anterior aspect of the ankle joint and the removal of this tissue is an adequate therapy to provide pain-free motion. Most of these devices use electromagnetic energy for shrinking, coagulation or ablation of tissue [3, 4]. In contrast, we used a bipolar device in which the energy comes from a plasma layer at the tip of the wand. Radio frequency (RF) ablation devices are widely used to remove soft tissue in arthroscopic surgery. The basic idea of a RF device is to destroy soft tissue by electrolyte plasmarization with the side effect of increased heating of the irrigation fluid. To avoid intraarticular heat, the RF device has vents that are inset at the tip to increase the outflow of the arthroscopic irrigation fluid. Unfortunately, some hot water, gas and denatured material may escape into the joint cavity where it might lead to unwanted increased local temperatures. Recent publications have shown dermal burns of patients due to hot water spilling originating from the RF ablation process and there are also some cases of glenohumeral chondrolysis after labral repair in hip arthroscopy [1–7]. Different reports investigated safety limits and found that chondrocyte damage may occur at temperatures as low as 45 °C/113 °F. Evaluations of the ability of the chondrocytes to recover showed a sharp increase of chondrocyte death between 50 to 55 °C (122 to 131 °F) [8, 9]. Thus, in recent publications on temperatures in shoulder joints [10] and hip joints [11], a 50 °C/122 °F criterion was used as limit for safe temperatures. Both publications report temperatures exceeding the critical point of 50 °C, which can easily be reached by RF ablation in the shoulder and hip joints depending on the flow of the irrigation fluid and their extraction. Until now, only limited data exists on the effects of RF ablation in the ankle joint, where especially the resection of hypertrophic synovia is useful and frequently used [12].

Anatomically, the capsular volume of the hip (2.5–10 ml) [13] is comparable to the volume of the ankle joint (6–10 ml) [14]. We therefore hypothesized that temperatures exceeding 50 °C/ (122 °F) may be reached by RF ablation. In our study, we wanted to investigate the impact of the irrigation flow rate, the controller unit voltage setting and the distance from the heat source on maximum temperatures, mean temperatures, time to reach the 50 °C/122 °F limit and – in view of the fact that the results of thermo-fluid dynamics experiments may vary substantially – the percentage of experimental runs exceeding 50 °C/122 °F with the same parameter settings.

## Methods

**Fig. 1:**
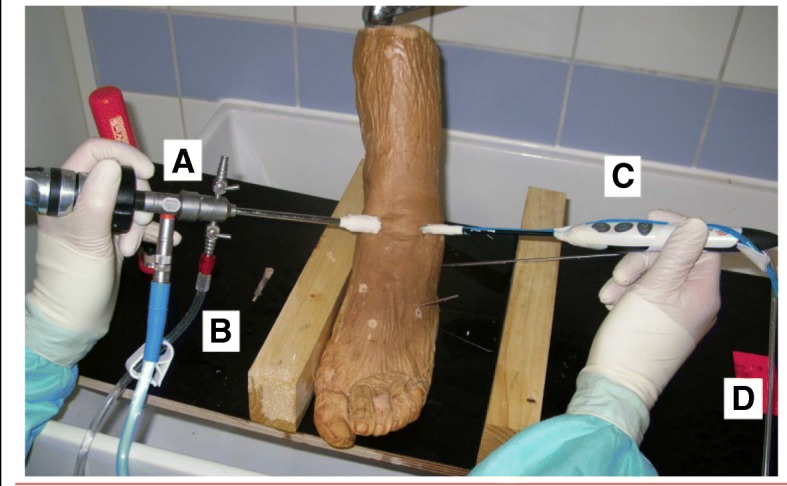
The laboratory setup used in this study with the arthroscope (A) and the irrigation hose (B) on the left side in the antero-medial portal and the bipolar radiofrequency device (C) in the antero-lateral portal. The thermometer probes are attached to the device. The radio frequency device was moved during the ablation process

A controlled laboratory study was designed using six formalin-fixed human cadaver ankle joints. All human specimen enrolled in this study were post-mortem donors to the Anatomical Institute of the University of Munich. The use of donated post-mortem specimens for scientific investigations is in accordance with the Declaration of Helsinki and was approved by the ethical committee of the University of Munich. Surrounding soft tissue around the ankle was preserved. The experiments were performed at room temperature using an antero-medial and antero-lateral portal. The irrigation inflow was placed in the antero-medial portal, while the bipolar RF device “Ambient Super Turbo Vac 90 IFS” with the Quantum II controller (Arthocare Corporation, Austin, Texas, USA) was placed via the antero-lateral portal (Fig. 1). The device uses a physical bipolar radio frequency process to stimulate electrolytes in the conductive natrium chloride solution. The energized particles in the plasma denaturize organic molecular bonds and dissolve tissue at temperatures between 40 °C and 70 °C/104 ° F and 158 °F, a current does not pass through the tissue. The result is volumetric removal of the target tissue with marginal collateral tissue damage. The RF device has vents at the tip, which are the outlets for the arthroscopic fluid. The Quantum II controller measures the temperature at the vents and cuts the power supply when temperatures exceed 40 °C/104 °F. Different voltage levels are possible, allowing a potential adaption according to the tissue treated.

**Fig. 2:**
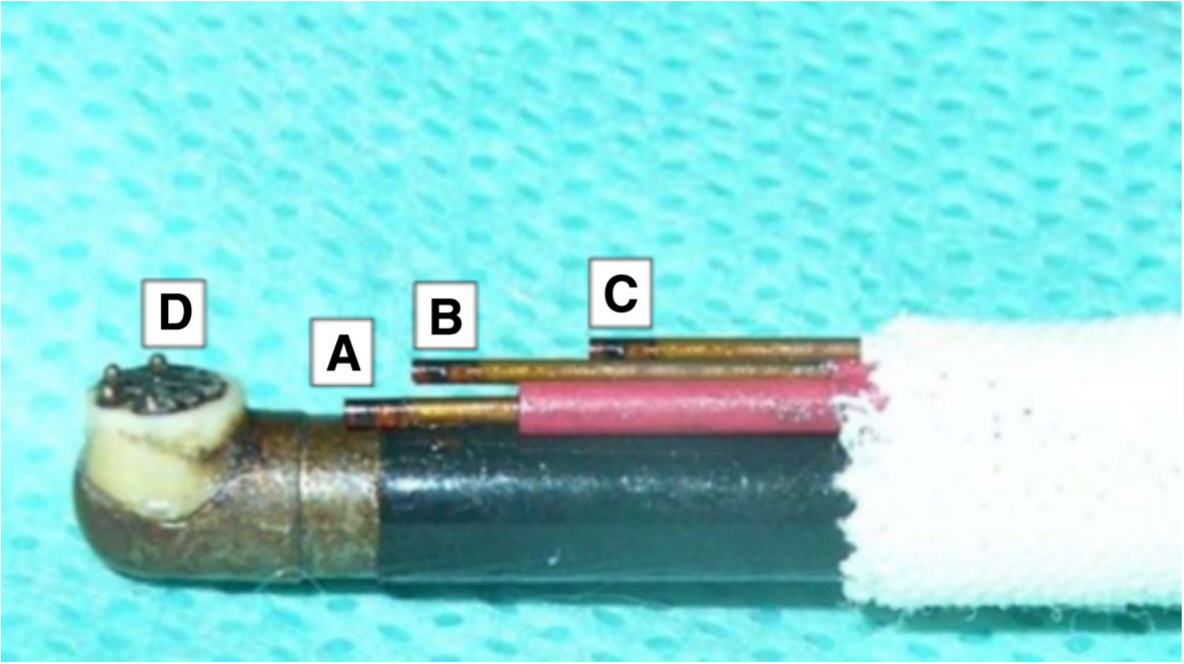
Three fiberoptical thermometer probes were attached to the radio frequency ablation device at 3 mm (A), 5 mm (B) and 10 mm (C) from the electrode plate with four ball electrodes where the heat is generated. The suction inlet consists of six triangular holes in this plate (D)

**Fig. 3:**
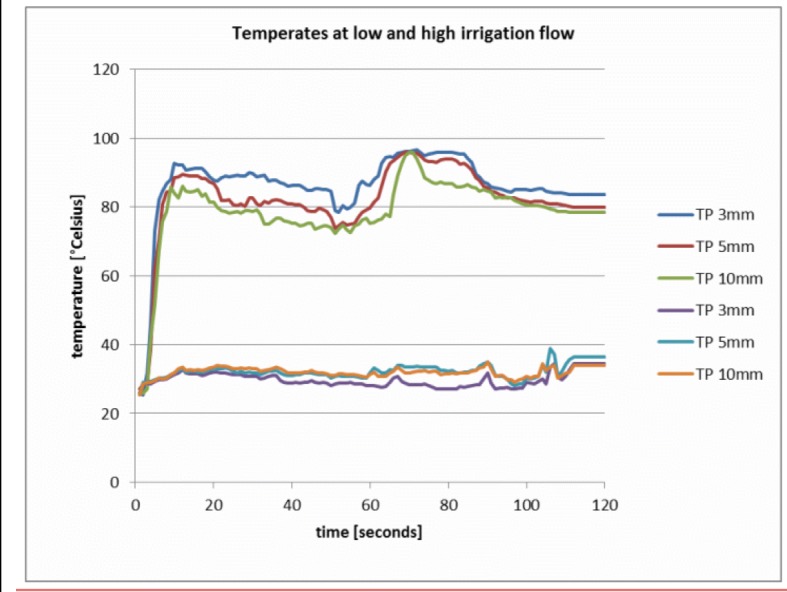
This figure shows temperature plots for an experiment with a pressure setting of 10 mmHg (three curves on the top) and with 100 mmHg (three bottom curves) for three thermometer probes at 3, 5 and 10 mm distance. For 10 mmHg, a rapid heat build-up is observed within the first 10 s. After the heat build-up, the temperature curves fluctuate around the average temperature. For 10 mmHg, the temperature fluctuations are more rapid and have smaller amplitudes than for 100 mmHg

All measurements were performed under direct arthroscopic vision, providing a free and unobstructed flow path for the irrigation fluid. To measure the temperature inside the ankle joint, three thermoprobes (TP) were placed 3, 5 and 10 mm off the tip (Fig. 2). Continuous temperature measurement was performed by a multichannel fiberoptical thermometer unit, which is sensitive enough to detect temperature changes of 0.01 °C. Temperatures were recorded and analyzed each second, while a measurement cycle took 120 s. In our view, the flow is the key parameter to excess heat in arthroscopy (Fig. 3). In order to vary the plasma power inside the cavity, the voltage was set from 1 to 5 and 9 at the end (minimum, medium and maximum level). The irrigation flow was controlled using the inlet pressures with 10, 25, 50 and 100 mmHg. The experiments started at room and irrigation fluid temperature of 20 °C/68 °F. During the 120 s, there is very little conductive heat flow from the cavity to the surrounding tissue and the heat capacity of the fluid in the ankle joint cavity is small (5–10 ml) (1). The experiments show that the heat escaping from the RF device leads to a heat build-up within 20 s at low coolant flow conditions. This clearly indicates that using 37 °C/98.6 °F instead of 20 °C/68 °F does not affect the maximum temperatures, but only reduces the time span before the stationary conditions are reached by a few seconds.

After having run four experiments for each combination as mentioned above, we analyzed our material and found that all experiments with the 100 mmHg pressure setting did not exceed the 50 °C/122 °F criterion, while the criterion was exceeded at least in one experiment for the other parameter combinations. However, we found no detectable effect of the voltage settings. Thus, the experiments with the same pressure setting are statistically in the same group, which means that we had 12 experiments for each pressure setting, which is sufficient for a meaningful statistical evaluation.

### Statistical analysis

Statistical analysis was performed using the software package SPSS™ (Version 19, IBM® Corporation, Somers, New York, USA) and the software EXCEL from Microsoft Office 2010 Professional. The temperature time histories were assessed and evaluated with EXCEL. Also, bounding curves and maxima and mean values were calculated with EXCEL. Linear regression analysis of the maximum and mean values was performed with both programs, SPSS and EXCEL. For the evaluation of the data, a *p*-value of 0.05 was assumed.

## Results

### Low, medium and high pressure temperature curves

In order to eliminate random effects, bounding curves were calculated for maximum temperatures for each pair of parameter settings for voltage and pressure position by averaging the TP positions and the four experimental runs. It was found that the maximum curves can be divided into three groups: low pressure (10 mmHg), medium pressure (25 and 50 mmHg) and high pressure (100 mmHg).

**Fig. 4:**
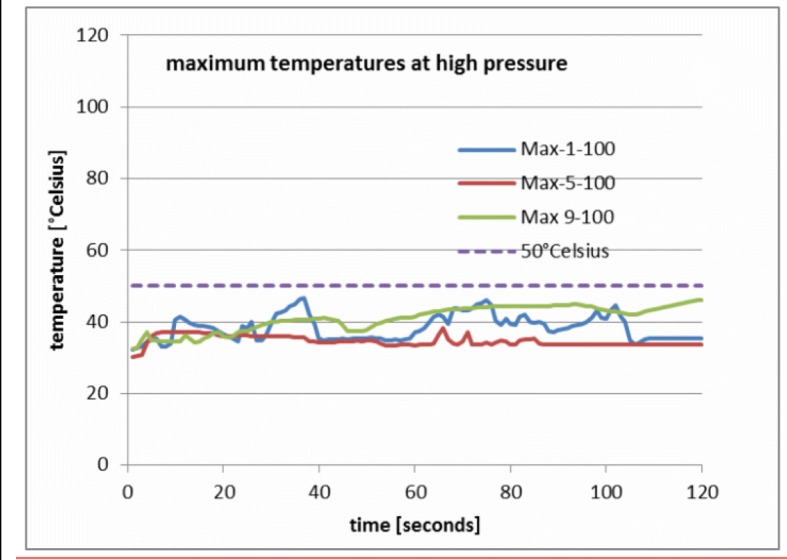
All maximum temperature curves for the different voltage settings 1, 5 and 9 and at low pressure of 10 mmHg are similar. Within a few seconds, the 50 °C criterion is exceeded and the temperature remains at high values of about 90 °C for the rest of the time

Figure 4 shows the resulting maximum curves for the low pressure setting. Without doubt, it is obvious that low pressure is not an option for ankle joint RF ablation - independent of the voltage setting.

**Fig. 5:**
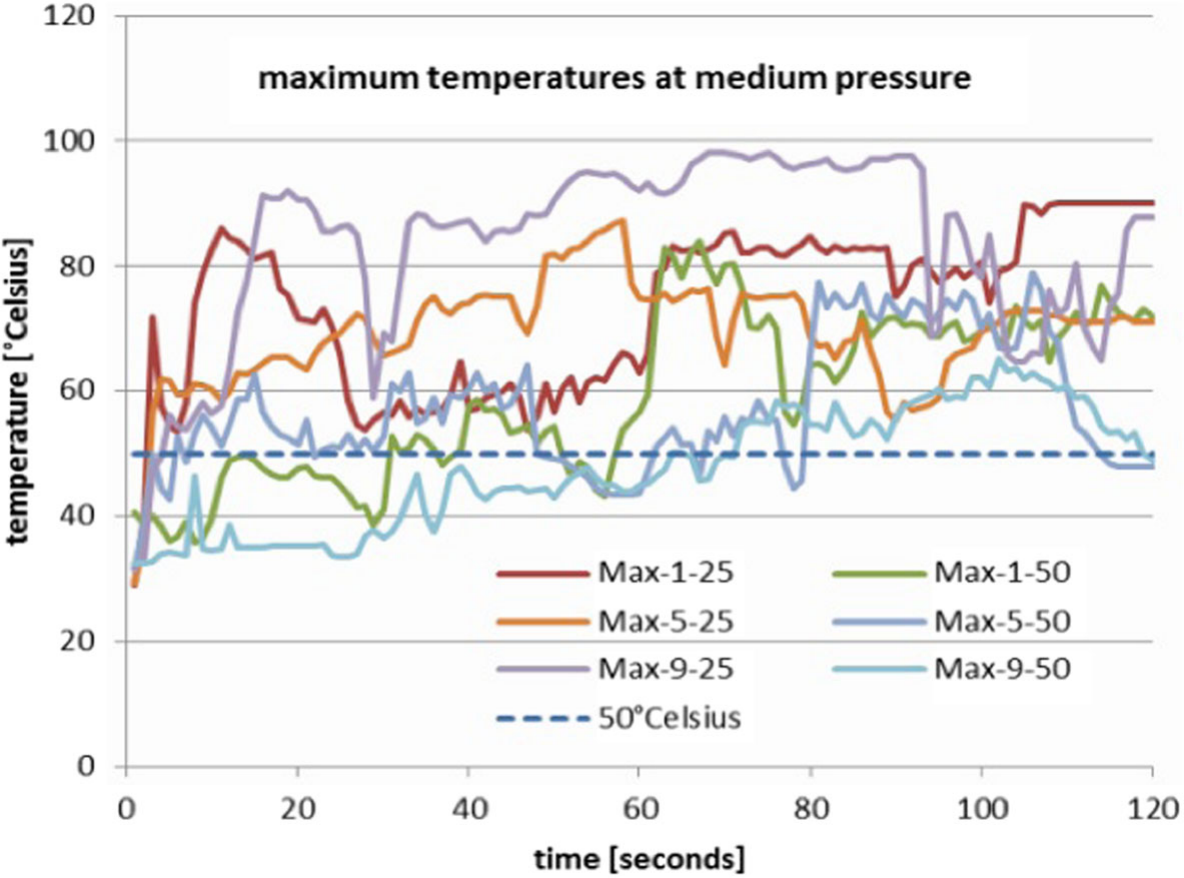
Maximum temperatures for voltage settings 1, 5 and 9 at medium pressure (25 and 50 mmHg). The curves show a strong oscillatory behavior and an upward tendency. All curves exceed the 50 °C criterion, lasting some seconds longer for curves with 50 mmHg

Figure 5 shows the resulting maximum curves for the medium pressure setting (25 and 500 mmHg). All curves show strong fluctuations and an overall upward tendency. All curves exceeded the 50 °C/122 °F criterion, for curves with 50 mmHg pressure, it takes some seconds longer. It is worse that the 50 °C/122 °F criterion is exceeded in all curves, if the time span is longer than 50 s in all cases. This means that an improved and more realistic version of the 50 °C/122 °F criterion is not feasible, e.g. a criterion like “surpassing the 50 °C/122 °F for a time span for less than 10 seconds”, in which no damage can be done because the time span is too short for a substantial heat build-up of the tissue.

**Fig. 6:**
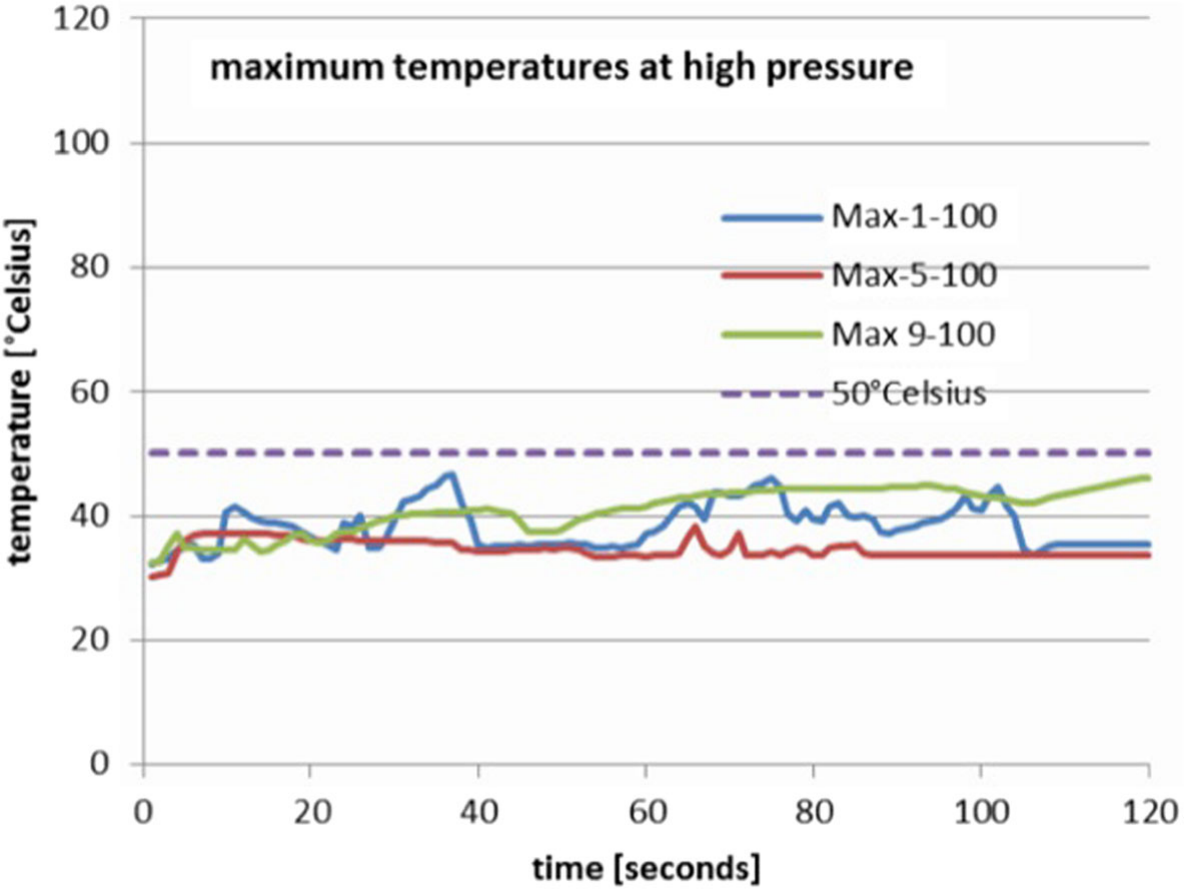
Maximum temperature for voltage settings 1, 5 and 9 at high pressure (100 mmHg). All curves remain below the 50 °C criterion

Figure 6 shows the resulting maximum curves for the high pressure setting (100 mmHg). These stay clearly below the 50 °C/122 °F criterion, show a stationary mean and only small fluctuation amplitudes. This indicates that using RF ablation with 100 mmHg is safe.

**Fig. 7:**
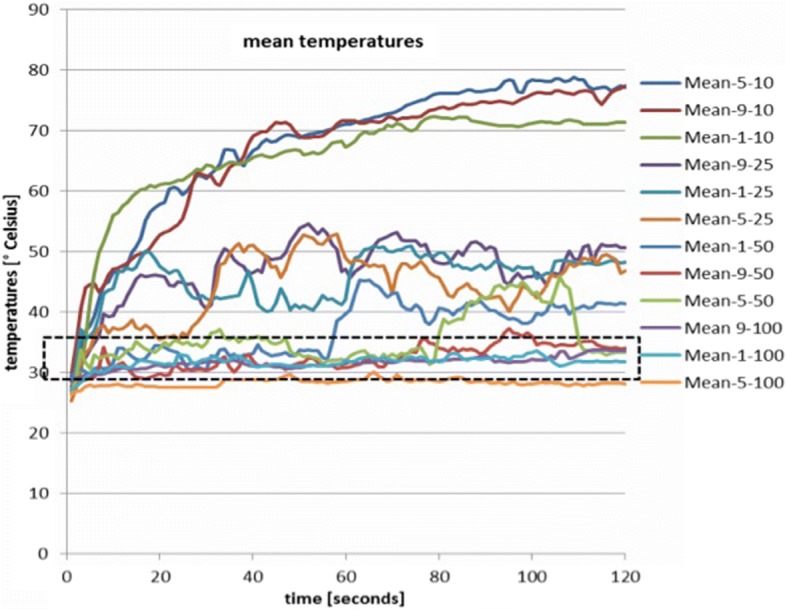
Mean temperatures for all voltage settings (1, 5 and 9) and all tested pressures (10, 25, 50 and 100 mmHg). The mean temperatures for the low pressure are substantially higher than the medium and high pressures. The range of mean temperatures at high pressure is marked by the dotted rectangle. Some of the temperatures at medium pressures stay within this rectangle for a limited amount of time, while others are clearly above this range all the time

Figure 7 shows the mean temperatures for all voltage settings (1, 5 and 9) and all pressures (10, 25, 50 and 100 mmHg). The mean temperatures for the low pressure setting are substantially higher than the medium and high pressures. The range of mean temperatures at high pressure is marked by dotted lines forming a rectangle. Some of the temperatures at medium pressures stay within the dotted lines for some time and then rise above the upper boundary, while others are clearly above this range most of the time. The overall behavior of the mean temperatures is similar to the overall behavior of the maximum temperatures as far as the influence of the different pressure settings is concerned.

### Regression analysis

In order to substantiate the expectations from a “quick look” analysis of the data and the physical interpretation of the process, regression analysis of the overall maximum and mean temperatures was carried out starting with the three parameters: voltage setting, square root of pressure and TP distance. Then, the parameter, which had the least effect was eliminated in two additional steps: in the second step, the voltage setting was eliminated and in the third step the TP distance. In a linear regression analysis, a multi-linear response surface is constructed that minimizes the distance of the measured data from the surface. The parameter R-squared is the proportion of variance of the maximum or mean temperature, respectively, which can be explained by the independent variables pressure, TP distance and voltage setting. We found that about 80% of the variances can be explained by the two variables pressure and TP distance. The *p*-value for the pressure is highly significant (*p* < 0.001), while the p-value for the TP distance is 0.011 for the mean temperature and 0.039 for the maximum temperature. This finding is in good compliance with the temperature fluctuations due to thermal convection, which may lead to a situation where the most distant TP sees higher temperatures than the TPs closer to the RF device with a certain probability. The p-value for the voltage setting is 0.856 for the mean and 0.818 for the maximum temperature, which means that any connection of the mean and maximum temperatures with the voltage setting is random. In summary, voltage setting did not have any impact on the maximum and mean temperatures and the key parameter to control the temperatures is pressure.

### Specific findings on individual experimental runs

Until now, we have presented findings on maximum and mean temperatures calculated by averaging the experimental runs. Next, the specifics of the individual runs will be addressed. It is characteristic for experiments in thermo-fluid dynamics in which some turbulence is generated that the temperature-time functions vary substantially from one experimental run to the next due to the dynamics of flow patterns and fluctuations. Running the same experiment with the parameters kept constant may result in temperature curves that differ substantially and fundamental aspects can only be identified by averaging various runs. A closer view on the individual violations of the 50 °C criterion illustrates this issue. In 100% of the low pressure experiments, the criterion was violated. In the medium pressure range for the 25 mmHg experiments, it was found that 66% of the experimental runs violated the criterion, while for the 50 mmHg runs only 38% violated the criterion. In the high pressure experiment, no violation was observed. Table 1 shows more details on the individual findings [11].

**Table 1:** Incidence of damaging temperatures reached Column 1 to 3 give the independent parameters. Column 4 lists the time needed to achieve temperatures above 50 °C. Column 5 lists the maximum temperatures observed in an individual experimental run

Parameters			Individual		Mean	
controller voltage setting	flow pressure [mm Hg]	Thermometer probe distance [mm]	first time ≥ 50 °C [_5_]	T_max_ [°C]	Percentage of runs with T_max_ ≥ 50 °C	Average Temp [°C] of all runs
1	10	3	4	96,5	100%	72,3
		5	5	96,0	75%	66,4
		10	5	95,8	75%	58,4
1	25	3	3	90,0	75%	52,7
		5	3	88,5	75%	47,6
		10	7	74,7	25%	36,6
1	50	3	31	47,6	40%	38,2
		5	58	48,2	40%	37,0
		10	58	46,4	20%	35,6
1	100	3	XX	46,6	0%	33,4
		5	XX	37,8	0%	31,9
		10	XX	38,5	0%	30,0
5	10	3	4	102,7	100%	77,0
		5	8	98,6	100%	65,7
		10	8	96,5	100%	60,3
5	25	3	3	87,2	0%	53,1
		5	30	87,4	25%	42,7
		10	37	74,2	50%	37,3
5	50	3	3	78,9	50%	39,6
		5	23	69,6	50%	36,9
		10	91	57,9	50%	30,8
5	100	3	XX	37,2	0%	29,6
		5	XX	38,3	0%	28,4
		10	XX	37,2	0%	27,1
9	10	3	2	99,6	100%	72,4
		5	3	100,5	75%	67,5
		10	20	92,3	75%	60,2
9	25	3	5	98,2	75%	55,6
		5	13	95,2	75%	49,8
		10	104	80,5	50%	36,2
9	50	3	72	58,6	25%	32,9
		5	64	46,2	25%	33,8
		10	XX	35,9	0%	30,5
9	100	3	XX	46,1	0%	33,1
		5	XX	40,4	0%	32,1
		10	XX	32,4	0%	29,9

#### The main findings presented in this table are

1. The shortest time span in which 50 °C/122 °F were reached was 2 s.
2. The highest maximum temperature was 102.7 °C (exceeding the boiling point of the saline fluid used as irrigation fluid) in experiments with low pressure and 32.4 °C/89.6 °F in experiments with high pressure.

3) The percentage of runs that exceed the criterion is an almost exact linear function of the square root of pressure.
4) The mean temperature ranges from 77.0 °C/170.6 °F at low pressure to 27.1 °C/80.6 °F at high pressure.

## Discussion

The purpose of this study was to investigate the effect of RF use in the ankle joint and how the three parameters irrigation fluid pressure, thermometer probe distance from the wand tip and the voltage setting of the controller affect the maximum temperatures in the ankle joint. The purpose was also to derive recommendations for the safe use of RF in the ankle joint.

There have been several reports about the deleterious effects of thermal damage to chondrocytes in shoulder arthroscopy resulting in chondrolysis [15, 16] and other papers indicate that a 50 °C/122 °F criterion should be observed since only temperatures below 50 °C/122 °F seem to be safe [9]. Two papers on the effect of RF use in the hip joint [11] and the shoulder joint [10] were published recently. Both papers found that temperatures in the joint, which exceed the criterion of 50 °C/122 °F, can be measured under no flow or low flow of irrigation fluid. From the analysis of the physical problem, it follows that the power output of the RF wand and the amount of irrigation flow are the key parameters, which influences the thermo-fluid dynamics. A typical phenomenon in thermo-fluid dynamics is temperature fluctuation. To measure these fluctuations close to the tip of the wand, three TPs were attached to the wand. From the physical analysis, it is also clear that there are many other parameters which may influence the temperature and which are not controlled in the experiment and therefore provide a random contribution to the data.

As the ankle joint volume (6–10 ml) is similar to the volume of the hip joint cavity (2.5–10 ml), we expected findings similar to those in the paper [10]. The regression analysis of the maximum temperatures (absolute maximum) and the mean temperatures (averaged over time) showed that 80% of the variances can be explained by the square root of the pressure (*p* < 0.001) and by the TP distance, which is also statistically significant (*p* < 0.05).

The effect of the voltage setting of the controller was random, which is hard to understand if one assumes that the voltage setting is relevant for the power output of the RF device. The maximum temperatures of the 12 runs at 100 mmHg were well below the 50 °C criterion, but for all other pressure settings, some or all runs clearly exceeded the criterion. It has to be concluded that high pressure has to be applied for safe temperatures. The physical analysis indicates that only the pressure difference across the cavity is relevant, thus an additional vacuum at the wand could be used to obtain the same thermo-fluid dynamics in the cavity with lower inlet pressure. The disadvantage of high pressures in the cavity is that water causes edema in the surrounding tissue. The situation can be improved by applying additional suction vacuum to reach the required pressure difference.

One option to reduce maximum temperatures in the ankle joint is the use of a turbulence-generating inflow opening at the arthroscope, which improves thermal mixing.

In addition, two issues were analyzed that might lead to even higher temperatures compared to those observed in this study: Hot non-condensable bubbles and outflow plugging. As the heat capacity of a non-condensable gas is lower than the heat capacity of water or tissue by orders of magnitude, it has to be concluded that hot non-condensable bubbles do not represent a potential hazard. As the RF device emits energized particles that break the molecular bounds within the tissue causing the tissue to dissolve, the ablation process generates more or less vapor and not particles, which might lead to plugging. Nevertheless, plugging cannot be ruled out as tissue may be cut loose and may be sucked into the RF device opening in the ablation process. Comparing the data found in our study and the studies on hip [10] and shoulder ablation [9], we could find similar temperature levels and no significant effect of the joint volume in view of the fact that the glenohumeral joint volume is more than twice the volume of the hip or the ankle joint [13, 14, 17].

Finally, the 50 °C/122 °F criterion is rather strict and does not reflect the chondrocyte damage mechanism. Obviously, a short contact with 50 °C will do no harm. Therefore, a better criterion that is based on more sophisticated and in-depth studies should be developed.

We deem the experimental settings of the study to be comparable to the surgical setting. We simulated the use of a RF device and were able to measure temperatures at the tip of the device directly. Although continuous use of the RF device for 60 s might be extreme, even a shorter time period (2 s) is able to cause cartilage damage. Therefore, it is tremendously important to ensure the correct irrigation and thereby diminish the risk of cartilage damage significantly.

In this context, it is crucial to keep in mind that not only high temperatures can have deleterious effects on the surrounding tissue, but also the use of increased water pressure. There have been reports about compartment syndromes of the lower leg after knee arthroscopy with injury to the posterior capsule [18] and of the anterior compartment after ankle arthroscopy in Maisonneuve fractures [19]. Mc Brayer et al. [20] could find an increase of swelling and deltoid muscle pressure during shoulder arthroscopy of 9 mmHg - particularly in operations lasting more than 90 min. Fortunately, they could not find a negative effect on the outcome in their study group.

Still, one has to think of the negative effects of increased pump pressures and what is more, Ross et al. [21] showed in their study that the operative field fluid pressure and the pressure readout might differ considerably.

There are limitations in this study: first, the use of cadaveric specimens leads to a different distraction of the ankle joint and the ankle joint cavity will differ in volume and shape affecting the flow pattern and fluctuations inside the cavity. Six cadaveric specimens cannot represent the full range of variability of human anatomy. Second, the procedure in this study with continuous ablation for more than 60 s may not reproduce the typical clinical scenario. Third, the cadaveric specimens were evaluated at room temperature and not at normal body temperature. Normal blood flow might work as a heat sink and dissipate heat. However, temperatures above the criterion of 50 °C/122 °F might be reached even faster at normal body temperature.

## Conclusions

This study investigated the temperatures in the ankle joint during RF ablation. It showed that the key parameter to guaranty temperatures below the 50 °C/122 °F criterion is irrigation pressure. At pressures of 100 mmHg, temperatures in the ankle joint were found to remain well below this criterion. At lower pressure levels, temperatures clearly exceeding 50 °C/122 °F were reached in some or all experiments. Until a more sophisticated in-depth study reveals more detailed information on the effect of RF ablation on the temperatures in the ankle joint and potential hazard for chondrocytes, the authors recommend to keep the pressure difference across the ankle joint as high as feasible, the ablation time short and the temperature of the irrigation fluid low.

## Abbreviations

RF: Radiofrequency
TP: Thermoprobe
